# Soluble Prokaryotic Overexpression and Purification of Bioactive Human Granulocyte Colony-Stimulating Factor by Maltose Binding Protein and Protein Disulfide Isomerase

**DOI:** 10.1371/journal.pone.0089906

**Published:** 2014-03-03

**Authors:** Bich Hang Do, Han-Bong Ryu, Phuong Hoang, Bon-Kyung Koo, Han Choe

**Affiliations:** Department of Physiology and Biomedical Institute of Technology, University of Ulsan College of Medicine, Seoul, Korea; New England BioLabs, United States of America

## Abstract

Human granulocyte colony-stimulating factor (hGCSF), a neutrophil-promoting cytokine, is an effective therapeutic agent for neutropenia patients who have undergone several cancer treatments. Efficient production of hGCSF using *E. coli* is challenging because the hormone tends to aggregate and forms inclusion bodies. This study examined the ability of seven different N-terminal fusion tags to increase expression of soluble hGCSF in *E. coli*. Four tag proteins, namely maltose-binding protein (MBP), N-utilization substance protein A, protein disulfide isomerase (PDI), and the b'a' domain of PDI (PDIb'a'), increased the solubility of hGCSF under normal conditions. Lowering the expression temperature from 30°C to 18°C also increased the solubility of thioredoxin-tagged and glutathione S-transferase-tagged hGCSF. By contrast, hexahistidine-tagged hGCSF was insoluble at both temperatures. Simple conventional chromatographic methods were used to purify hGCSF from the overexpressed PDIb'a'-hGCSF and MBP-hGCSF proteins. In total, 11.3 mg or 10.2 mg of pure hGCSF were obtained from 500 mL cultures of *E. coli* expressing PDIb'a'-hGCSF or MBP-hGCSF, respectively. SDS-PAGE analysis and silver staining confirmed high purity of the isolated hGCSF proteins, and the endotoxin levels were less than 0.05 EU/µg of protein. Subsequently, the bioactivity of the purified hGCSF proteins similar to that of the commercially available hGCSF was confirmed using the mouse M-NFS-60 myelogenous leukemia cell line. The EC_50_s of the cell proliferation dose-response curves for hGCSF proteins purified from MBP-hGCSF and PDIb'a'-hGCSF were 2.83±0.31 pM, and 3.38±0.41 pM, respectively. In summary, this study describes an efficient method for the soluble overexpression and purification of bioactive hGCSF in *E. coli*.

## Introduction

Granulocyte colony-stimulating factor (GCSF), also known as pluripoietin, controls the production, differentiation, and function of granulocytes, which account for 70% of white blood cells [1], [2]. The recruitment of two monomers of GCSF triggers dimerization of the GCSF receptor and initiates a signaling cascade [3]–[5]. Production of GCSF, which is secreted predominantly by macrophages, fibroblasts and endothelial cells, is stimulated by several inflammatory stimuli, including interleukin-1β, tumor necrosis factor-alpha, and lipopolysaccharide [6]–[8]. Human GCSF (hGCSF) has been approved for the treatment of neutropenia, a common disorder in cancer patients following radiotherapy or chemotherapy treatments, characterized by an extremely low number of neutrophils in the blood [9], [10]. GCSF also has neuroprotective properties [11]; accordingly, the protein has been used as a protective agent in mouse models of various neurodegenerative diseases, including amyotrophic lateral sclerosis [12], [13].

Human GCSF was initially purified from a tumor cell line that continuously secreted the protein [14]. When expressed in the methylotrophic yeast *Pichia pastoris*, hGCSF is secreted in a soluble form; however, the secreted protein is highly aggregated and must be solubilized using high concentrations of denaturants such as guanidine hydrochloride or urea. Consequently, purification of the biologically active form of hGSCF from yeast requires the removal of these denaturants and refolding of the protein [15]. *Escherichia coli* also produces aggregated hGCSF in inclusion bodies (IBs) [16]–[22]; however, the overall yield of biologically active protein from these structures is usually low [23]. Alternatively, hGCSF can be secreted into the periplasm of *E. coli* [24], [25], although low yields are also usually obtained using this method. Maltose-binding protein (MBP), and stress-responsive proteins such as peptidyl-prolyl cis-trans isomerase B, bacterioferritin, and glutathione synthase, have previously been tested as fusion partners to increase the production of solubilized hGCSF in *E. coli* [26], [27].

In this study, several new methods of overexpressing soluble hGCSF in the cytoplasm of *E. coli* were investigated, enabling efficient production of biologically active protein. The following seven N-terminal fusion tags were used: hexahistidine (His6), thioredoxin (Trx), glutathione S-transferase (GST), MBP, N-utilization substance protein A (NusA), protein disulfide bond isomerase (PDI), and the b'a' domain of PDI (PDIb'a'). The MBP, NusA, PDI, and PDIb'a' tags increased the solubility of hGCSF markedly at 30°C. Lowering the expression temperature to 18°C also increased the solubility of Trx- and GST-tagged hGCSF, whereas His6-hGCSF was insoluble at both temperatures. The expression level and the solubility of the tag-fused hGCSFs were also tested in the *E. coli* Origami 2(DE3) strain that have mutations in both the thioredoxin reductase (trxB) and glutathione reductase (gor) genes, which may assist the disulfide bond formation in the cytoplasm of *E. coli* [28]–[30]. Simple methods of purifying hGCSF from the PDIb'a' or MBP tagged proteins were developed using conventional chromatographic techniques. In total, 11.3 mg of biologically active hGCSF was obtained from 500 mL of culture. Silver staining indicated that the extracted hGCSF was highly pure and the endotoxin level was very low. The activity of the purified protein was measured using a bioassay with mouse M-NFS-60 myelogenous leukemia cells.

## Materials and Methods

### Construction of plasmids and expression in *E. coli*

**Figure 1:**
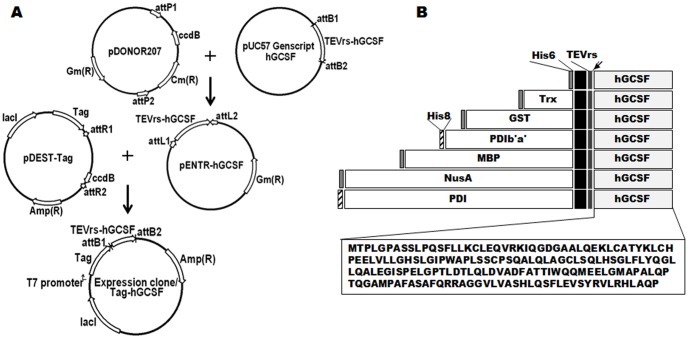
Construction of the hGCSF expression vectors and schematic representations of the domain structures. A. The method of construction and vector map of the tag-hGCSF construct. All fusion constructs were generated in the same way via LR recombination cloning. Expression of the fusion proteins in *E. coli* was controlled by the IPTG-inducible T7 promoter, and ampicillin was used as the selection marker. B. Schematic representation of the seven hGCSF fusion proteins used in this study (His6-, Trx-, GST-, PDIb'a'-, MBP-, PDI-, and NusA-hGCSF). The arrow indicates the TEV protease cleavage site. Black is extra sequences from BP and LR recombinations. The amino acid sequence of mature hGCSF is also shown.

The h*GCSF* gene (Uniprot identifier: P09919-2) encodes a protein comprising 204 amino acids, the first 29 of which form the signal peptide. To enable the expression and purification of hGCSF in *E. coli*, a tobacco etch virus (TEV) protease recognition site (TEVrs; ENLYFQˇG) was appended to the N-terminus of mature hGCSF (175 amino acids), and two site-specific recombination sequences, attB_1_ (5′-GGGGACAAGTTTGTACAAAAAAGCAGGCTTC-3′) and attB_2_ (5′-ACCCAGCTTTCTTGTACAAAGTGGTCCCC-3′), were added to each end of the gene sequence (Figures 1A and 1B). The h*GCSF* DNA sequence which is substituted Met^1^ to Ala^1^ was synthesized and subcloned into plasmid pUC57 (Genscript, Piscataway, NJ), which was then recombined with the pDONOR207 vector (Invitrogen, Carlsbad, CA) to produce the entry vector pENTR-hGCSF (Figure 1A). LR recombination cloning between pENTR-hGCSF and seven destination vectors containing the relevant fusion tags (pDEST-HGWA, pDEST-HXGWA, pDEST-HGGWA, pDEST-HMGWA, pDEST-HNGWA, pDEST-PDI, and pDEST-PDIb'a') [31], [32] was performed to produce expression vectors containing tagged hGCSF. The expression plasmids were confirmed by DNA sequencing (Macrogen, Daejeon, Korea) and then transformed into *E. coli* BL21(DE3) and Origami 2(DE3).

To overexpress hGCSF, the transformed BL21(DE3) cells were grown at 37°C in 200 rpm of shaking incubator in 2 mL of Luria-Bertani (LB) broth containing 50 µg/mL ampicillin. For the culture of the transformed Origami 2(DE3), 12.5 µg/mL tetracycline was also added. One mM isopropyl-β-D-thiogalactoside (IPTG) was added at 0.4∼0.6 OD_600_ to induce the expression of the hGCSF fusion proteins. The cells were harvested after incubation for 5 h at 30°C or 12 h at 18°C.

### Purification of hGCSF from the PDIb'a'-hGCSF fusion protein

*E. coli* BL21(DE3) cells transformed with the PDIb'a'-hGCSF expression vector were cultured for 12 h at 18°C in 500 mL of LB medium. When OD_600_ was reached to 0.4∼0.6, 1 mM IPTG was added to induce the expression of the fusion protein. The collected cells were resuspended in 50 mL of immobilized metal ion affinity chromatography (IMAC) binding buffer comprising 50 mM Tris-HCl (pH 8.0), 500 mM NaCl, and 5% glycerol (v/v). The solution was sonicated until completely transparent and then centrifuged for 20 min at 27,000 g to generate the supernatant. After equilibrating with binding buffer, the pre-packed 3×5 mL HisTrap HP column (GE Healthcare, Piscataway, NJ) was fed with the lysate solution and non-specific proteins were then removed by washing with IMAC buffer containing 100 mM imidazole. The PDIb'a'-hGCSF fusion protein was eluted in IMAC buffer containing 500 mM imidazole. To support TEV protease cleavage, the buffer was then exchanged to NaCl-free IMAC buffer (50 mM Tris-HCl, pH 8.0, 5% glycerol (v/v)) using a dialysis membrane (Viskase, Darien, Illinois). For digestion, the fusion protein was incubated with TEV protease at a ratio of 1∶20 for 12 h at 18°C. For IMAC, the digested sample was loaded onto a pre-packed 2×5 mL HisTrap HP column filled with IMAC buffer. Unlike other proteins in solution, hGCSF had a low affinity to the Ni resin and was easily eluted from the HisTrap column using IMAC buffer containing 50 mM imidazole. Based on the chromatogram, the collected hGCSF was analyzed by 10% Tris-tricine SDS-PAGE.

### Purification of hGCSF from the MBP-hGCSF fusion protein

*E. coli* BL21(DE3) cells transformed with the MBP-hGCSF expression vector were cultured for 12 h at 18°C in 500 mL of LB medium and induced by 1 mM IPTG when OD_600_ was 0.4∼0.6. Due to the high affinity of MBP-hGCSF to the MBP column, a 2×5 mL MBPTrap HP column (GE Healthcare) was used as the first purification step. The cells were resuspended in 50 mL of MBP-binding buffer comprising 50 mM Tris-HCl (pH 8.0), 0.5 mM EDTA, 200 mM NaCl, and 5% glycerol (v/v), and then sonicated to form a soluble solution. The supernatant was loaded onto a 2×5 mL MBPTrap HP column equilibrated with MBP-binding buffer. Non-specific bound proteins were removed by washing with binding buffer and MBP-hGCSF was eluted with binding buffer containing 10 mM maltose monohydrate. The eluted sample was diluted until the final concentration of NaCl was 50 mM and then cleaved with TEV protease under the same conditions as described for PDIb'a'-hGCSF. Cleaved hGCSF was then purified using the same method of hGCSF cleavage from PDIb'a'-hGCSF.

### SDS-PAGE and silver staining

Proteins were separated and visualized on a 10% Tris-tricine gel stained with Coomassie Brilliant Blue R-250 (AMRESCO, Solon, OH). The expression, solubility, and purity were quantified using ImageJ software (http://imagej.nih.gov/ij). For silver staining, the polyacrylamide gel was placed into Fixative Enhancer Solution (Bio-Rad Laboratories, Hercules, CA) for 20 min and then rinsed with distilled water to increase the sensitivity and contrast of the staining. Staining and developing were performed using a mixture of silver complex solution, reduction moderator solution, and image development reagent (Bio-Rad Laboratories, Hercules, CA). The reaction was stopped by the addition of 5% acetic acid.

### Endotoxin assay

To remove endotoxins from purified hGCSF, the solution was incubated with 1% Triton X-114 (Sigma-Aldrich, St. Louis, MO) at 4°C for 30 min. Triton X-114 was accumulated after incubating the sample at room temperature and removed by centrifugation at 9,000 g for 10 min [33]. The Endpoint Chromogenic Limulus Amebocyte Lysate test (Lonza, Basel, Switzerland) was used to quantify the remaining endotoxin in the target solution. Briefly, Limulus Amebocyte Lysate was incubated with the hGCSF sample at 37°C for 10 min before the substrate was added. Stop agent (25% v/v glacial acetic acid) was then added to the mixture and the released p-nitroaniline was evaluated by photometric measurement at 405–410 nm.

### Cell proliferation assay

The M-NFS-60 mouse myelogenous leukemia cell line [34], [35], kindly provided by Dr. Kyung-Woon Kim (Rural Development Administration, Suwon, Korea), was grown in RPMI-1640 medium (Invitrogen, Carlsbad, CA) containing 10% fetal bovine serum, 1X penicillin and streptomycin (Invitrogen), and 0.05 mM β-mercaptoethanol [36]. The cells were maintained at 37°C in a humidified atmosphere containing 5% CO_2_. The bioassay of purified hGCSF using M-NFS-60 cells was based on the 3-(4,5-dimethylthiazol-2-yl)-2,5-diphenyltetrazolium bromide (MTT) assay (Invitrogen). The cultured cells were seeded at a density of 3×10^4^ cells/well into 96-well plates containing growth medium. To determine its effect on proliferation of the cells, different concentrations (0.1, 1, 10, 100, 1,000, 10,000 and 100,000 pg/mL) of commercially available hGCSF purified from IB (Genscript, Piscataway, NJ) and hGCSF produced from the PDIb'a' and MBP fusion proteins were added to each well in a final volume of 100 µL. After 72 h of incubation, 15 µL of 5 mg/mL MTT was added to each well and the cells were incubated in the dark at 37°C for a further 4 h. After removing all solutions from the cells, 100 µL of dimethyl sulfoxide was added to each well to completely solubilize the formed aggregates. The optical density of the solution was measured at 570 nm using an ELISA plate reader (Molecular Devices, Sunnyvale, CA).

### Data analysis

A non-linear regression analysis was used to determine the M-NFS-60 cell proliferation dose-response to hGCSF. The data were fitted using the following equation and Microsoft Excel software, where *Re* is response of the cells, *Bl* is the baseline at low concentration, *Max* is the maximum response, *conc* is the concentration of the protein, and *Hs* is the Hill coefficient of stimulation, *Bh* is the baseline at high concentration, and *Hi* is the Hill coefficient of inhibition:
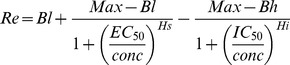
(1)


All data are presented as the mean ± standard error (SE) of n≥3 of 2 independent experiments. To determine the statistical significance of the responses of cells to hGCSF, group means were compared using a Student's *t*-test or a one-way analysis of variance followed by Bonferroni's multiple comparisons test. Graphpad Prism 5 software (GraphPad, San Diego, CA) was used for statistical analyses and *P*<0.05 was considered significant.

## Results

### Construction of plasmids and expression of tagged hGCSF in *E. coli*

To enable soluble expression of hGCSF in the cytoplasm of *E. coli*, the following seven tags were fused to the N-terminus of the protein via LR recombination cloning: His6, Trx, GST, PDI b'a', MBP, PDI, and NusA (Figure 1). A TEVrs was also inserted between each tag and hGCSF to facilitate removal of the tags during purification, and the sequence was codon-optimized for *E. coli* expression (Figure 1B). Vectors containing the fusion tags were recombined with the hGCSF plasmid, then the resulting plasmids were sequence-verified and transformed into the BL21(DE3) *E. coli* strain, which lacks protease expression.

**Figure 2:**
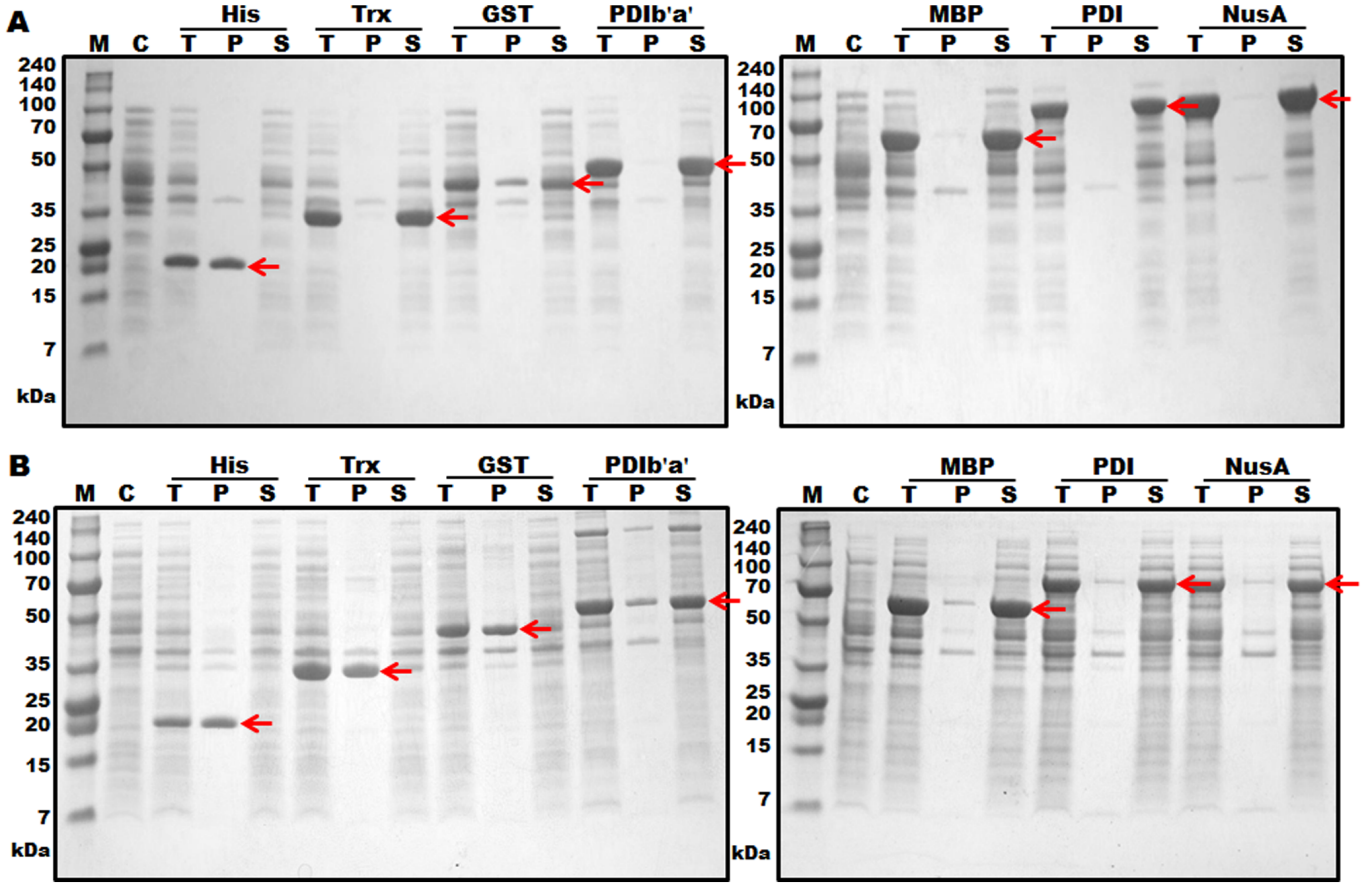
Expression levels of hGCSF fused with seven different tags in *E. coli* BL21(DE3). Protein expression was induced with 1°C (A) or 30°C (B). After sonication, 20 µg of each total protein was loaded onto a 10% Tris-tricine gel. The arrows indicate the hGCSF fusion proteins. M, molecular weight size marker; C, total protein before IPTG induction (control); T, total protein after IPTG induction; P, protein in the cell pellet after sonication; S, protein in the supernatant after sonication.

Expression of the h*GCSF* fusion genes in *E. coli* was controlled by a T7 promoter and induced with 1 mM IPTG at two different expression temperatures of 30°C and 18°C. The expression levels of all tagged hGCSF proteins were 33–68%, and the expression levels of all proteins were higher at 18°C than 30°C (Figure 2 and Table 1). The solubilities of the proteins varied depending on both the type of fusion tag used and the expression temperature. The solubility of hGCSF at 30°C was markedly enhanced by the addition of the MBP, NusA, PDI, and PDIb'a' tags (Figure 2B and Table 1). Lowering the expression temperature to 18°C additionally increased the solubility of the Trx-hGCSF and GST-hGCSF proteins to similar levels (Figure 2A and Table 1); however, His6-hGCSF was insoluble at both expression temperatures. We also tested *E. coli* Origami 2(DE3), a strain that may promote disulfide bond formation in the cytoplasm of *E. coli*, as an expression host. The expression levels of the fusion proteins in Origami 2(DE3) were lower than those in BL21(DE3), and the solubilities were similar at both 18°C and 30°C (Figure S1). Based on the expression level, solubilities and sizes of the tagged proteins, PDIb'a'-hGCSF and MBP-hGCSF in BL21(DE3) were selected for further study.

**Table 1:** Expression levels and solubilities of hGCSF fused with seven different N-terminal tags.

	Tag	Tag size (kDa)	Fusion protein size (kDa)	Expression (%)		Solubility (%)	
				18°C	30°C	18°C	30°C
hGCSF (18.8 kDa)	His6	0.8	23.5	43.8	33.6	-	-
	Trx	11.8	35.3	61.4	48.8	98.3	5.0
	GST	25.7	49.2	41.3	40.0	78.4	3.2
	PDIb'a'	35.6	59.1	66.3	42.2	96.0	73.5
	MBP	40.3	63.8	61.4	58.4	96.5	88.1
	PDI	55.1	78.7	55.6	43.8	98.1	89.3
	NusA	54.9	78.4	68.0	44.8	97.5	89.5

### Purification of hGCSF from the PDIb'a'-hGCSF fusion protein

**Figure 3:**
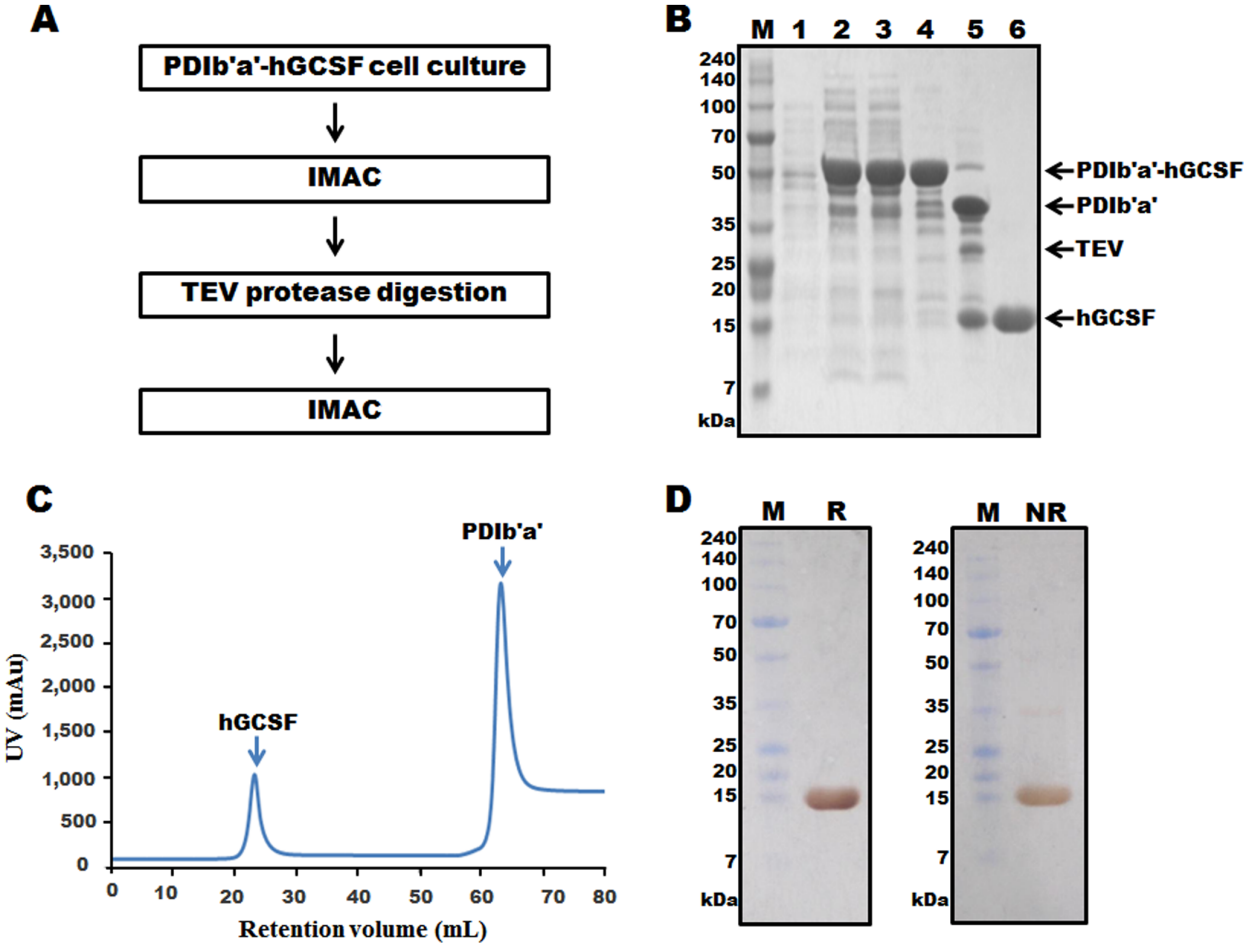
Purification of hGCSF from the PDIb'a'-hGCSF fusion protein expressed in *E. coli* BL21(DE3). A. Overview of the purification process using IMAC chromatography and TEV protease digestion. B. SDS-PAGE analysis of hGCSF at various stages of the purification process. Ten micrograms of total protein was loaded onto a 10% Tris-tricine gel. M, molecular weight marker; lane 1, total protein before IPTG induction; lane 2, total protein after IPTG induction; lane 3, soluble fraction after cell sonication; lane 4, PDIb'a'-hGCSF fusion protein purified by the first round of IMAC (59.1 kDa); lane 5, PDIb'a'-hGCSF fusion protein following TEV protease cleavage showing separated PDIb'a' (35.6 kDa) and hGCSF (18.8 kDa); lane 6, purified hGCSF after the second round of IMAC (18.8 kDa). C. IMAC chromatogram of the TEV protease cleaved PDIb'a'-hGCSF fusion protein showing clear separation of the PDIb'a' tag and hGCSF. D. Silver staining of 7.5 µg of purified hGCSF. M, molecular weight marker; R, reduced hGCSF; NR, non-reduced hGCSF.

Separation of hGCSF from the PDIb'a'-hGCSF fusion protein was performed by two rounds of IMAC, with an intervening TEV protease digestion step (Figure 3A). IMAC was possible because all of the tags used in the study contained an additional His6 or His8 tag at their N-terminal end (Figure 1B). Cells transformed with the plasmid containing PDIb'a'-hGCSF were induced with IPTG and then collected (Figure 3B, lane 2). The cells were lysed and centrifuged to harvest the supernatant (Figure 3B, lane 3), which was then loaded onto a Ni column and the binding protein was eluted after a washing step (Figure 3B, lane 4). Most of the non-specific proteins were removed at this step; however, some minor contaminant bands were observed. Despite the presence of these additional proteins, TEV protease digestion was performed. After optimizing the digestion conditions (data not shown), the majority of the PDIb'a'-hGCSF protein was cleaved by TEV protease (Figure 3B, lane 5). A second HisTrap HP column was then used to remove the PDIb'a' tag, undigested PDIb'a'-hGCSF, and TEV protease, which also contained a His6-tag. Cleaved hGCSF weakly bound to the Ni column and was eluted by 50 mM imidazole (Figure 3C). An SDS-PAGE analysis revealed the absence of any contaminating proteins after this step (Figure 3B, lane 6). Silver staining of the SDS-PAGE gel under reducing and non-reducing conditions showed that the purified hGCSF protein was highly pure and mostly monomeric (Figure 3D). Typically, 11.3 mg of hGCSF was obtained from a 500 mL culture of *E. coli* expressing PDIb'a'-hGCSF, with a yield of 36.7% (Table 2). After treatment with Triton X-114, the endotoxin level of hGCSF purified from the PDIb'a'-hGCSF fusion protein was 0.05 EU/µg.

**Table 2:** The characteristics of hGCSF purified from PDIb'a'-hGCSF and MBP-hGCSF fusion proteins expressed in *E. coli*.

Purification step	hGCSF purified from PDIb'a'-hGCSF				hGCSF purified from MBP-hGCSF			
	Total protein(mg)	Purity (%)	hGCSF(mg)	Yield (%)	Total protein(mg)	Purity (%)	hGCSF (mg)	Yield (%)
Cell weight	1500	-		-	1500	-		-
Supernatant	140	69.1	30.8	100	118.8	75.9	26.6	100
1^st^ Chromatography (IMAC/MBP)	71.5	73.3	16.7	54	79.8	88	20.7	77.8
2^nd^ Chromatography	11.4	99	11.3	36.7	10.3	99	10.2	38.3

### Purification of hGCSF from the MBP-hGCSF fusion protein

**Figure 4:**
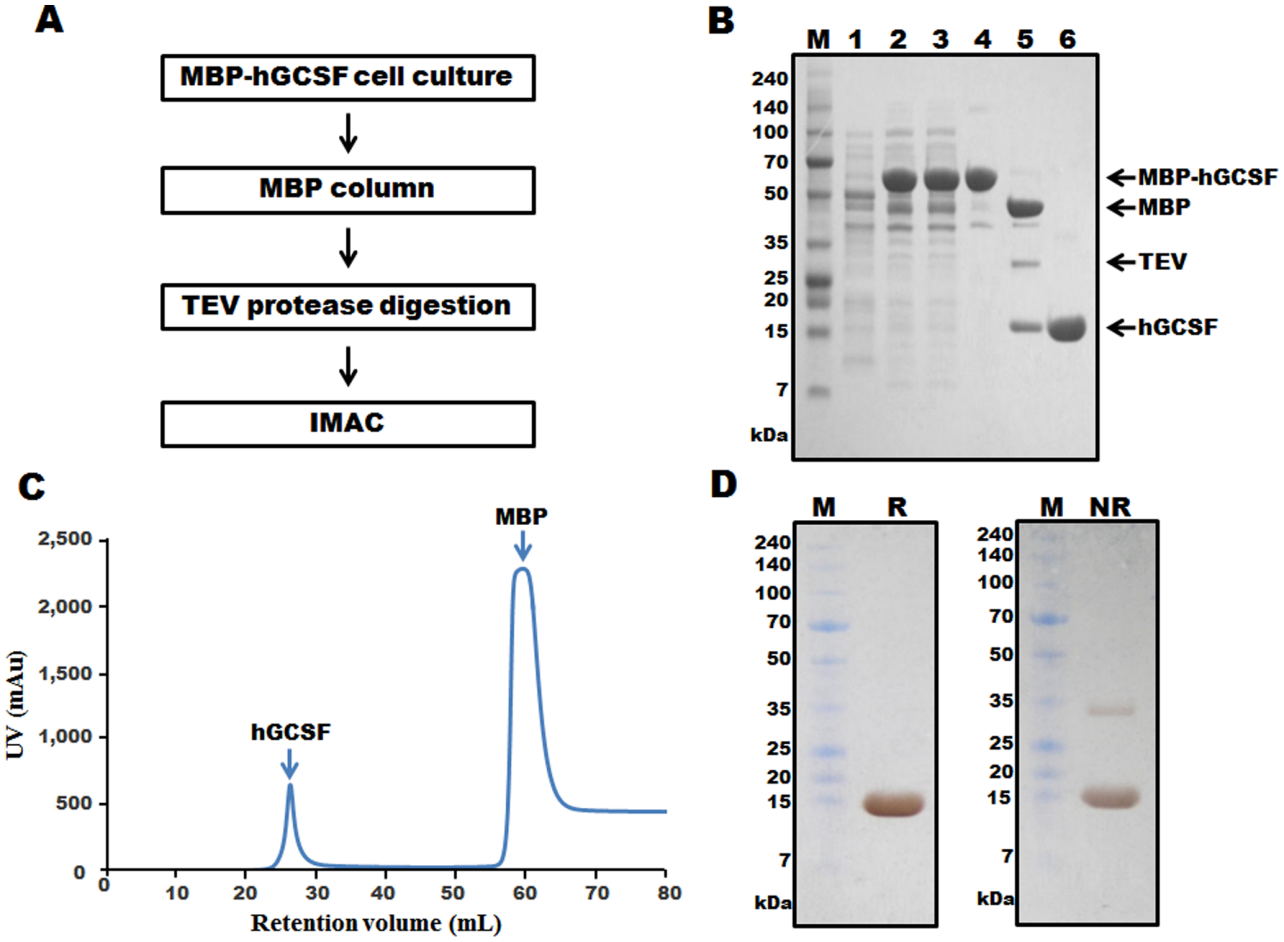
Purification of hGCSF from the MBP-hGCSF fusion protein expressed in *E. coli* BL21(DE3). A. Overview of the purification process using an MBP affinity column, TEV protease digestion, and IMAC. B. SDS-PAGE analysis of hGCSF at various stages of the purification process. M, molecular weight marker; lane 1, total protein before IPTG induction; lane 2, total protein after IPTG induction; lane 3, soluble fraction after cell sonication; lane 4, MBP-hGCSF fusion protein purified using an MBP affinity column (63.3 kDa); lane 5, MBP-hGCSF fusion protein after cleavage with TEV protease showing separated MBP (40.3 kDa) and hGCSF (18.8 kDa); lane 6, purified hGCSF after IMAC (18.8 kDa). C. IMAC chromatogram of the TEV protease cleaved MBP-hGCSF fusion protein showing clear separation of the MBP tag and hGCSF. D. Silver staining of 7.5 µg of purified hGCSF. M, molecular weight marker; R, reduced hGCSF; NR, non-reduced hGCSF.

Figure 4A shows an outline of the process used to purify hGCSF from MBP-hGCSF in the cell lysate. MBP chromatography isolated the MBP-hGCSF fusion protein from the total protein mixture with a purity of approximately 80% (Figure 4B, lane 4). After cleavage of the fusion protein with TEV protease (Figure 4B, lane 5), the sample was applied to a Ni-NTA column and purified hGCSF was obtained by eluting with 50 mM imidazole (Figure 4B, lane 6; Figure 4C). Similar to the highly pure hGCSF (approximately 99%) obtained from PDIb'a'-hGCSF, silver staining of the SDS-PAGE gel under reducing and non-reducing conditions revealed the presence of highly pure hGCSF isolated from MBP-hGCSF (Figure 4D). Most of the purified protein was monomeric; although a small amount of hGCSF dimer was observed under non-reducing conditions (Figure 4D). Typically, 10.2 mg of purified hGCSF was obtained from a 500 mL culture of MBP-hGCSF. This total yield of 38.3% (Table 2) was lower than from PDIb'a'-hGCSF. After treatment with Triton X-114, the level of endotoxin in the purified hGCSF sample was 0.013 EU/µg. The endotoxin level of bio-products is typically less than 1 EU/µg.

### Biological activity of hGCSF

**Figure 5:**
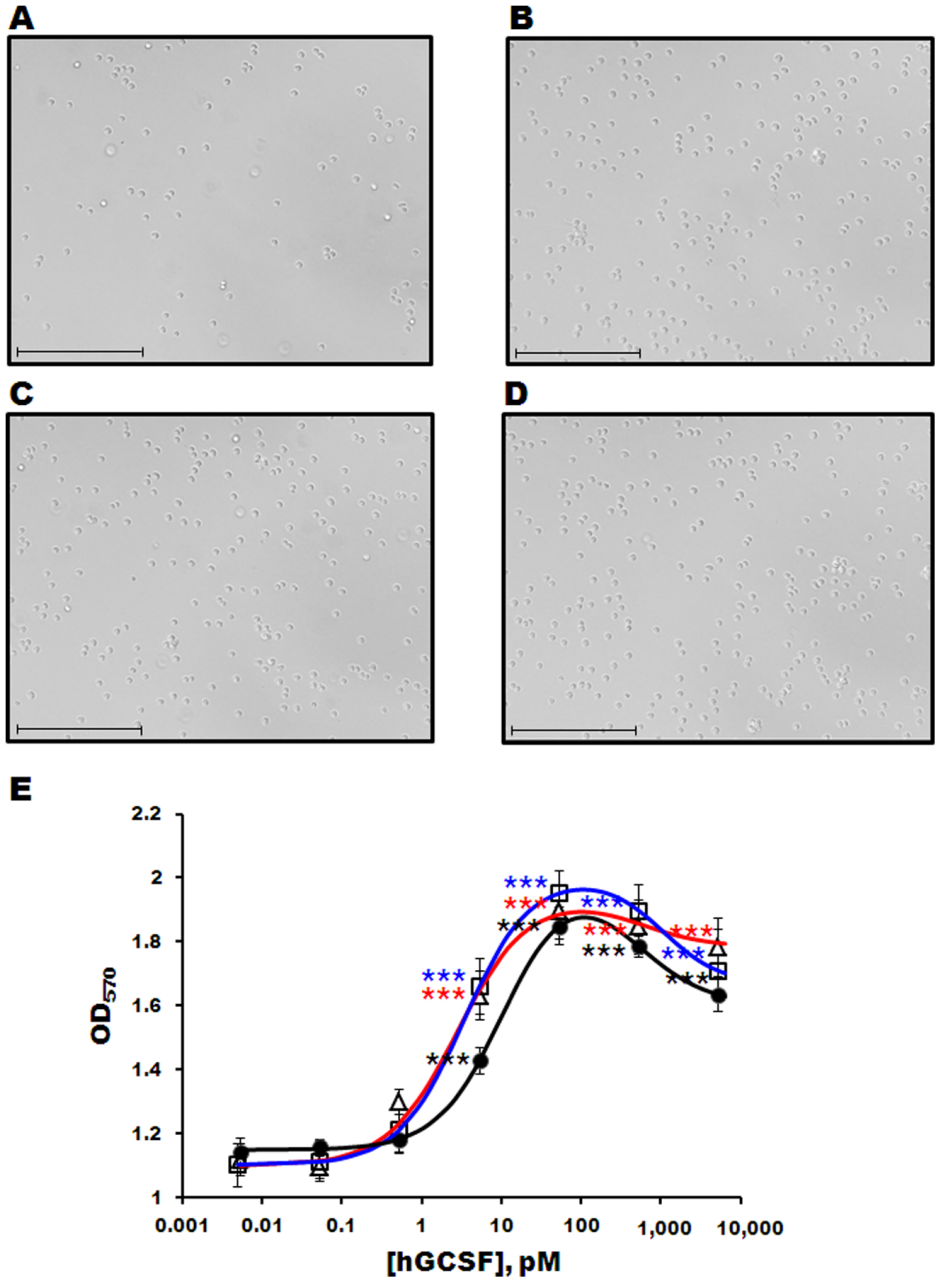
Cell proliferation assay of purified hGCSF using the M-NFS-60 cell line. Light microscopy images of M-NFS-60 cells incubated without (A) or with (B–D) 1 ng/mL hGCSF for 48 h. Commercially available hGCSF (B), hGCSF purified from MBP-hGCSF (C), and GCSF purified from PDIb'a'-hGCSF (D) were used. The scale bar represents 100 µm. E. Dose-response curve of M-NFS-60 cells following exposure to different concentrations of purified hGCSF and commercial hGCSF. The number of cells was measured as the OD_570_. •, commercially available hGCSF; ▵, hGCSF purified from MBP-hGCSF; hGCSF purified from PDIb'a'-hGCSF. Data are represented as the mean ± SE of n≥3 of 2 independent experiments. Statistical significance compared to no hGCSF treatment group: *, p<0.05; **, p<0.01; ***, p<0.001.

The bioactivities of the purified hGCSF proteins were measured using an MTT assay and the mouse M-NFS-60 myelogenous leukemia cell line. The number of M-NFS-60 cells increased dramatically after incubation with commercially available hGCSF or hGCSF purified from the PDIb'a'-hGCSF or MBP-hGCSF fusion proteins (Figures 5A–E). At concentrations below 1 nM, the dose-response curves were sigmoidal for all three forms of hGCSF (Figure 5E); however, higher concentrations produced mild inhibition, resulting in a bell-shaped curve (Figure 5E). The EC_50_s of commercial hGCSF, hGCSF from MBP-hGCSF, and hGCSF from PDIb'a'-hGCSF were 10.69±2.62 pM, 2.83±0.31 pM, and 3.38±0.41 pM, respectively, with Hill coefficients of 1.06±0.29, 1.00±0.05, and 1.06±0.11, respectively. The differences between the EC_50_s and Hill coefficients were not statistically significant, suggesting that the hGCSF proteins purified from MBP-hGCSF and PDIb'a'-hGCSF are as slightly better effective as commercially available hGCSF.

## Discussion

Many human proteins expressed in prokaryotes such as *E. coli* are prone to accumulation in IBs. Consequently, time-consuming solubilization and refolding are necessary to generate the purified proteins; processes that are also hampered by low yields, poor reproducibility, and the generation of proteins with low biological activity [23], [37]. When expressed in *E. coli*, hGCSF is also insoluble, and so to address this problem, this study examined the effect of seven different fusion tags that function as chaperones, as well as the effect of a low expression temperature, on the solubility of hGCSF.

The MBP, PDI, PDIb'a', and NusA tags solubilized greater than 70% of the hGCSF fusion protein at 30°C, whereas the solubilities of the Trx-, GST-, and His6-tagged proteins were low at this temperature (Table 1, Figure 2). MBP is thought to act as a general molecular chaperone [38] by binding to hydrophobic residues present on protein surfaces [39]. MBP-tagged proteins can be easily purified with commercially available MBP-binding columns. PDI forms and breaks disulfide bonds of proteins in the lumen of the endoplasmic reticulum. The cytoplasm is usually a reducing environment that prevents proper disulfide bond formation, but PDI increases the production of soluble proteins in both the cytoplasm [40] and periplasm of *E. coli* [41]. PDI is composed of four thioredoxin-like domains, named a, b, b', and a'. The a and a' domains display redox-active catalytic and chaperone activities, whereas the b and b' domains only demonstrate some chaperone functions [42]. Previous experiments in our laboratory have shown that PDIb'a' increases the solubility of several proteins to the same degree as PDI (data not shown); however, the data presented here show that PDIb'a' was less effective than PDI at solubilizing hGCSF. NusA was suggested as a solubilizing tag protein based on the revised Wilkinson-Harrison solubility model [43], [44], which predicted NusA to be 95% soluble and to improve the solubility of several proteins. PDI and PDIb'a' were also predicted to be good solubilizing agents according to this model (data not shown). The revised Wilkinson-Harrison solubility model considers the number of four turn-forming residues (Asn, Gly, Pro, and Ser) and determines the net charge by subtracting the number of acidic residues from the number of basic residues. However, this model may have some limitations because it predicted relatively low solubility for the MBP, Trx, and GST tags (data not shown), despite the fact that hGCSF fused with these tags showed good solubility.

With the exception of His6-hGCSF, lowering the expression temperature from 30°C to 18°C increased the solubility of all tagged hGCSF proteins (Table 1, Figure 2). Low expression temperatures have been successfully used in the past to increase the solubility of many proteins expressed in *E. coli* [45]–[50]; however, the molecular mechanisms responsible for this effect are not fully understood at present. The cold temperature protein chaperones are induced at low temperatures [51]; peptidyl-prolyl isomerase is a known cold temperature protein chaperone that catalyzes cis/trans isomerization of the peptide bonds found in proline residues [52]. In addition, several ATP-consuming heat shock proteins may also play a role in improving protein solubility at low expression temperatures [53]. Although highly inducible by heat shock treatment, these proteins are expressed at normal temperatures and have chaperone functions. However, the effects of lowering the expression temperature on protein solubility cannot be generalized because His6-tagged hGCSF was not soluble at all at 18°C.

The effects of hGCSF purified from MBP-hGCSF or PDIb'a'-hGCSF on the proliferation of M-NFS-60 cells were slightly higher than that of commercially available hGCSF (Figure 5E). The EC_50_ values for hGCSF purified from MBP-hGCSF (2.83 pM) and PDIb'a'-hGCSF (3.38 pM) were consistent with a previous study that reported an EC_50_ value in the range of 0.8–6 pM for hGCSF [25], [54], [55]. At high concentrations, the purified hGCSF proteins induced mild inhibition of cell proliferation, resulting in a bell-shaped biphasic dose-response curve (Figure 5E). This is consistent with a previous report that other cytokines also show a biphasic dose-response curve [56].

There are three splicing variants of hGCSF. The short isoform (b) used in this study is reportedly more active than the longer isoform (a) [57], and the third isoform lacks the region spanning amino acids 37 to 73. In this study, we substituted the first amino acid (Ala) with Met, and this mutation increased binding of hGCSF to its receptor [58] and facilitated PEGylation of the N-terminus of the protein, which increased the half-life of GCSF in blood [59].

Mature hGCSF contains five cysteine residues, four of which form two native intramolecular disulfide bonds, Cys^37^-Cys^43^ and Cys^65^-Cys^75^. A previous study in which Cys^18^ was mutated to Ser demonstrated that Cys^18^ is not required for bioactivity of hGCSF [60]. However, during folding of hGCSF, intermolecular disulfide bonds between two Cys^18^ residues or Cys^18^ and another Cys residue can occur in aggregates [61]. The formation of subsequent dimers or multimers can render hGCSF insoluble in *E. coli* cytoplasm. As a result of the non-optimal spatial orientation of the molecules, the activity of the GCSF dimer is much lower than that of the GCSF monomer *in vitro* [62]. Some effective solutions, such as the mutation of Cys^18^ [21], [36] or the addition of a specific secretory signal peptide that directs the secretion of hGCSF into the periplasmic space [24], have been used to overcome this obstacle in *E. coli*. Here, soluble monomeric hGCSF with bioactivity similar to that of hGCSF purified from HEK cells was obtained using a fusion protein strategy and a low expression temperature.

Mature hGCSF is glycosylated at Thr^134^. One limitation of using *E. coli* to produce hGCSF is the lack of glycosylation machinery in the bacterial cells; therefore, overexpressed hGCSF obtained from *E. coli* is non-glycosylated. Glycosylation prevents protein aggregation and increases the half-life of circulating proteins in the blood by protecting proteins from protease cleavage; however, it does not affect the binding of proteins to receptors. Indeed, the clinical effects of glycosylated and non-glycosylated hGCSF on chemotherapy-induced neutropenia were not significantly different statistically in a clinical trial [63].

## Conclusion

This study demonstrates that fusion proteins and a low expression temperature can be used to successfully express soluble hGCSF in the cytoplasm of *E. coli*. Using simple chromatographic techniques and TEV protease digestion, >10 mg of highly bioactive hGCSF was purified from 500 mL cultures of cells expressing MBP-hGCSF or PDIb'a'-hGCSF.

## Supporting Information

Figure S1 **Expression levels and solubilities of hGCSF fused with seven different tags in** ***E. coli*** **Origami 2(DE3).** Protein expression was induced with 1 mM IPTG at either 18°C (A) or 30°C (B). After sonication, 20 µg of each total protein was loaded onto a 10% Tris-tricine gel. The arrows indicate the hGCSF fusion proteins. M, molecular weight size marker; C, total protein before IPTG induction (control); T, total protein after IPTG induction; P, protein in the cell pellet after sonication; S, protein in the supernatant after sonication.
(TIF)
