# Supplementary figures and images for: Soluble Prokaryotic Overexpression and Purification of Bioactive Human Granulocyte Colony-Stimulating Factor by Maltose Binding Protein and Protein Disulfide Isomerase
Source: PLoS One. 2014 Mar 3;9(3):e89906. doi: 10.1371/journal.pone.0089906 (PMC3940694; doi:10.1371/journal.pone.0089906)

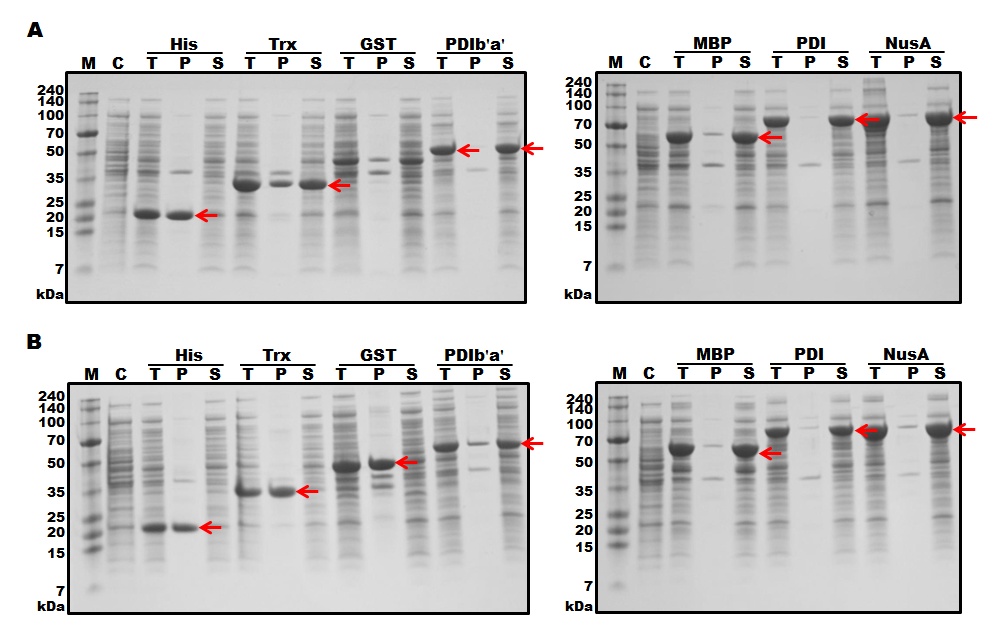

Supplement: Figure S1 — Expression levels and solubilities of hGCSF fused with seven different tags in E. coli Origami 2(DE3). Protein expression was induced with 1 mM IPTG at either 18°C (A) or 30°C (B). After sonication, 20 µg of each total protein was loaded onto a 10% Tris-tricine gel. The arrows indicate the hGCSF fusion proteins. M, molecular weight size marker; C, total protein before IPTG induction (control); T, total protein after IPTG induction; P, protein in the cell pellet after sonication; S, protein in the supernatant after sonication. (TIF) [file pone.0089906.s001.tif]
